# *Flux-Based* Transport Enhancement as a Plausible Unifying Mechanism for Auxin Transport in Meristem Development

**DOI:** 10.1371/journal.pcbi.1000207

**Published:** 2008-10-31

**Authors:** Szymon Stoma, Mikael Lucas, Jérôme Chopard, Marianne Schaedel, Jan Traas, Christophe Godin

**Affiliations:** 1 Virtual Plants Project-Team, UMR DAP, INRIA, Montpellier, France; 2 Laboratoire de Reproduction et Développement des Plantes, Université de Lyon 1, INRA, CNRS, ENS-Lyon, Lyon, France; University of Virginia, United States of America

## Abstract

Plants continuously generate new organs through the activity of populations of stem cells called meristems. The shoot apical meristem initiates leaves, flowers, and lateral meristems in highly ordered, spiralled, or whorled patterns via a process called *phyllotaxis*. It is commonly accepted that the active transport of the plant hormone auxin plays a major role in this process. Current hypotheses propose that cellular hormone transporters of the PIN family would create local auxin maxima at precise positions, which in turn would lead to organ initiation. To explain how auxin transporters could create hormone fluxes to distinct regions within the plant, different concepts have been proposed. A major hypothesis, *canalization*, proposes that the auxin transporters act by amplifying and stabilizing existing fluxes, which could be initiated, for example, by local diffusion. This convincingly explains the organised auxin fluxes during vein formation, but for the shoot apical meristem a second hypothesis was proposed, where the hormone would be systematically transported towards the areas with the highest concentrations. This implies the coexistence of two radically different mechanisms for PIN allocation in the membrane, one based on flux sensing and the other on local concentration sensing. Because these patterning processes require the interaction of hundreds of cells, it is impossible to estimate on a purely intuitive basis if a particular scenario is plausible or not. Therefore, computational modelling provides a powerful means to test this type of complex hypothesis. Here, using a dedicated computer simulation tool, we show that a *flux-based* polarization hypothesis is able to explain auxin transport at the shoot meristem as well, thus providing a unifying concept for the control of auxin distribution in the plant. Further experiments are now required to distinguish between *flux-based* polarization and other hypotheses.

## Author Summary

Plants continuously generate new organs through the activity of populations of stem cells called meristems. The shoot apical meristem (SAM) initiates leaves, flowers, and lateral organs in highly ordered, spiraled, or whorled arrangements via a process called phyllotaxis. Auxin, a plant hormone, plays an essential role in this process. It is actively transported from cell to cell by specific membrane-associated transporters. In the SAM, this coordinated transport creates organized auxin fluxes resulting in hormone accumulation at precise positions, where organ formation is triggered. One key question in this process is to understand how auxin transport is coordinated. To address this issue, we have investigated a specific hypothesis, the canalization hypothesis, whereby every cell senses and attempts to stabilize existing hormone fluxes. Because such a patterning process would require the interaction of hundreds of cells, it is impossible to estimate on a purely intuitive basis whether it would be able to generate the observed organ positions. We, therefore, developed a computational approach to test this hypothesis and showed that a flux-based mechanism is indeed able to generate phyllotactic patterns and is consistent with biological data describing meristem development.

## Introduction

During plant development, organs are continuously created by small populations of cells called *apical meristems*. The so-called shoot apical meristem (SAM) generates all the aerial parts of the plant. The SAM is a highly organized structure, composed of a central zone required to maintain the meristem and a surrounding peripheral zone, that is competent to initiate new organ primordia [1]. The young organs are usually initiated in highly ordered spiralled or whorled patterns. This remarkable arrangement of organs is called phyllotaxy and varies according to particular plant species and growth conditions. Over the last two centuries, phyllotaxy has been extensively studied and different models for this patterning process have been proposed. From a mechanistic point of view, it is now widely accepted that phyllotaxy emerges from a process of local lateral inhibition: each primordium creates an inhibitory field in its vicinity where no other primordium can develop. This basic inhibitory field hypothesis (see [2] for a review), is potentially able to generate a wide range of phyllotactic patterns [2]–[6].

Hypotheses concerning the physiological nature of these inhibitory fields were proposed only recently [7]. They rely on the signalling role of a key hormone called *auxin*, that plays a crucial role in primordium formation [1]. Auxin is actively transported throughout the plant from cell to cell by carriers that are located at the cell plasma membranes [8]. During auxin transport influx carriers of the AUX/LAX family, facilitate auxin import into the cells. This is in contrast to the PIN-FORMED (PIN) proteins, which facilitate efflux [9]. Interestingly, PIN proteins often accumulate on one particular side of the cell, thus suggesting that auxin is evacuated preferentially via that side. Importantly, PIN carriers often show locally coherent orientations between groups of neighbouring cells, indicating that PIN orientation is coordinated at the level of tissues [7],[10]. It is therefore possible to imagine how cells could transport auxin from cell to cell throughout the plant, thereby creating fluxes that lead to local hormone maxima and minima [11],[12]. These differences in concentration would subsequently be interpreted in terms of differential gene expression and growth rates.

At the SAM, auxin importers and exporters are mainly expressed at the meristem surface [7]. When auxin transport is inhibited, organ initiation is severely affected or even totally absent [13]. In addition, the cells at the SAM orient their PIN proteins towards the young primordia and it is now currently thought that organs are initiated at auxin accumulation points, while the hormone is depleted in their neighbourhoods [7]. The young primordia would thus create drainage basins in their vicinity which would be equivalent to the inhibitory fields proposed previously. While the coherent behaviour of PIN proteins in cell populations is well established, the actual mechanism behind this phenomenon is still not well understood. So far two basic concepts have been proposed.

A first hypothesis is based on the pioneering work of Sachs (1969) on vascular tissue differentiation in plants [14]. Sachs proposed that auxin transport is facilitated during the process of vascular tissue induction. He suggested that the positive feedback between flux and transport is able to amplify small fluxes and can potentially create *canals* of auxin between auxin sources and sinks that subsequently differentiate into vein tissues. This positive feedback between flux and transport is at the basis of the *flux-based* polarization mechanism we study in this work. The *canalization* hypothesis was then formalized by Mitchison [15],[16] who developed a mathematical model of this process that increases membrane permeability of cell plasma membrane on the sides where the net flux of auxin is positive. This model was then further studied in the context of leaf venation pattern by several authors [17]–[20] who interpreted the *canalization* hypothesis as a feedback mechanism between auxin fluxes and PIN transporters and studied the properties of such a coupling both on a fixed shape and during leaf development. From the biological point of view, recent experiments tend to support the *canalization* hypothesis, at least in the inner tissues of the plant [21]–[23]. However, whether it could also account for the behaviour of auxin transporters in other parts of the plant such as the shoot and root apical meristem or leaf margins remains an open question.

**Figure 1:**
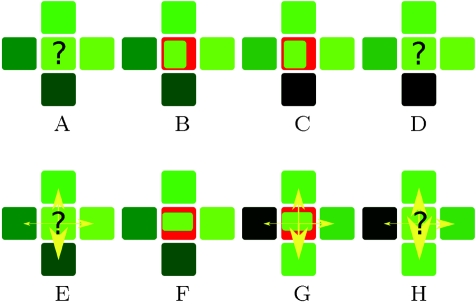
Comparison of two PIN orientation hypotheses. The concentration of auxin in the cells is marked with green (the brighter green, the higher the concentration), the fluxes are depicted with yellow arrows, and PIN concentration at the membranes are indicated by red lines with variable thickness (the thicker the line the higher the concentration). Note that the fluxes might be independent from the concentrations. (E–H) show the principle of the *concentration-based* hypothesis and (A–D) show the principle of *flux-based* polarization hypothesis. In both cases the key question is how the cell marked with “?” should allocate the PINs to its membranes (A,E). In the case of the *concentration-based* hypothesis this cell makes the decision based on the *concentrations* in the neighbouring cells. The higher its neighbours auxin concentration, the more PIN will be inserted in the membrane (F). *Flux-based* polarization depends on the *net flux* between neighbouring cells. The higher the *net flux* to its neighbour the more PIN will be inserted in the membrane (B). In both cases the newly allocated PINs change the concentrations and fluxes (C,G), leading to the next iteration of the scenario (D,H).

More recently, a second concept was proposed to explain auxin transport at the SAM surface [24],[25]. Based on the observation that PIN carriers point to primordia initiation sites in the SAM which supposedly correspond to auxin maxima, it was hypothesized that relative concentrations of auxin in neighbouring cells differentially drive the polarization of PIN1 to the corresponding portion of the membrane between each cell and its neighbours [24]. The cells would thus tend to export auxin against the auxin concentration gradient (referred to here as *concentration-based* hypothesis), thus amplifying differences in local auxin concentrations and creating *local maxima* or *spots* of auxin [22]. The comparision of *concentration-based* and *flux-based* polarization hypothesis is presented in Figure 1, Using computational modelling, several authors were able to show that *concentration-based* hypothesis can produce spiralled and whorled phyllotactic patterns. In a recent article, Merks et al. proposed a modified *concentration-based* hypothesis [26]. Although it requires further development, it is potentially able to explain the formation of veins in internal tissues. Could it, therefore, represent a unifying mechanism for the control of auxin fluxes throughout the plant? A major argument against this idea is that the model does not seem to be compatible with the presence of stable auxin maxima in tissues. This is typically the case at the root meristem, where a continuous, stable auxin maximum is maintained with incoming and outgoing fluxes [27]. In a recent study, Sauer et al. suggested that cell-type specific factors could decide whether one or the other mechanism would be used [23], but this remains to be proven.

Since the *concentration-based* hypothesis can not, on its own, provide a unifying mechanism for the control of auxin fluxes in the plant, we investigated whether *flux-based* polarization hypothesis, as the other major concept in the field could provide a realistic alternative. Since such cell-cell signalling based patterning processes involve the interactions between hundreds of cells it is impossible to estimate on a purely intuitive basis if a particular scenario is plausible or not. In this context, computational modelling provides a powerful means to test this type of hypotheses. We therefore designed a set of models and showed that the *flux-based* polarization mechanism is able to:

1. generate spiral phyllotactic patterns observed in the SAM,
2. produce provascular strands below primordia in the subepidermal meristem layers,
3. reproduce stable auxin maxima as observed in the root meristems.

We therefore conclude that flux-based polarization could provide a unifying principle for the guidance of auxin fluxes in the plant. In addition, our model leads to a set of testable predictions, that should be able to distinguish between the *flux-based* and *concentration-based polarization* hypotheses.

## Models

### Biological Assumptions

To model auxin transport in a tissue we used a set of auxin related hypotheses derived from biological observations taken from the literature (see also introduction):

1. **The Auxin quantity in a cell changes as a result of active transport and diffusion between cells and local creation/degradation** [28], as in previous models [10],[24],[25].
2. **Auxin is created locally in every cell** (suggested by Reinhard et al. [29], also used in other models [17],[24],[25]). At this stage all precise locations of auxin synthesis is not well defined, but several of the YUCCA genes involved in auxin synthesis are expressed at the shoot meristem [30].
3. **Auxin is degradated locally in every cell**, e.g., see [31].
4. Auxin is transported from the cell into the inter-cellular space according to the chemiosmotic model [32]. Briefly, this supposes that it is difficult for auxin to leave the cell by diffusion because of the neutral pH of the cytoplasm, whereas it can enter it more freely from the acidic inter-cellular space. Therefore, the plant has developed a system of transporters that facilitates the transport from cell to cell [8],[33]. At the meristem, only PIN transporters seem to be polarized, while the AUX/LAX influx carriers are homogeneously distributed over the membrane. We model this overall transport process using a simplified system. First, we assume direct flux of auxin from cell to cell by omitting the wall compartment. Second, due to the symmetry of influx carriers, only PIN is simulated explicitly. Therefore, **we model auxin redistribution in the meristem as a result of passive diffusion between cells and polar transport which is governed by PIN**. A similar approach was also used in other transport models [10], [17], [24]–[26].
5. **PIN concentration in a cell membrane is up-regulated by auxin flux through this membrane** [14]. This hypothesis is explained in detail in the Mathematical Formalization section.

To design the model of phyllotaxis, we extended the auxin related hypotheses with a set of hypotheses related to phyllotaxis:

1. The shoot apical meristem is a dome shaped structure, containing up to thousands of cells. **We distinguish the epidermal layer, called L1, that is one cell thick from the subepidermal cells that makes up the rest of the dome.**
2. **The L1 layer is itself composed of a central zone surrounded by a peripheral zone (also called competence zone)**. These zones exhibit different properties [34] as explained below.
3. **Primordia can appear only in the peripheral zone of the meristem** [**1**]. Once a primordium is initiated, it moves away from the meristem summit following a radial trajectory, due to cell growth throughout the L1 [2],[35].
4. **In the L1, primordium cells act as sinks by redirecting auxin from the L1 layer downwards**. This hypothesis is justified by the presence of vascular strands below each primordium which would transport auxin downwards [7]. We assume that a primordium can easily remove any amount of auxin (the saturation level is much higher than the amount of auxin available in meristem).
5. Longitudinal sections show that provascular strands are approximately three cells wide (data not shown). Therefore we assume that **a primordium is constructed from a central cell and all its direct neighbours**.
6. **A new primorium is formed as a response to high auxin accumulation in a cell of the competence (peripheral) zone** [1].
7. **Auxin and PIN reallocation are fast processes. PIN proteins can be reallocated within one or two hours (** [**23**], **our own unpublished results). Growth occurs at a slower timescale. Typically, a cell doubles its volume in 24 h** [36]. Therefore, as a simplification, we consider auxin concentrations and PIN localisation to be in equilibrium at the time scale used to model growth.
8. **Auxin is concentrated in the L1 and accesses the inner layers via primordia**. Because of the presence of **AUX/LAX** auxin importers, it has been proposed that auxin is concentrated in the L1 layer. It is mainly transported to the inner tissues via the provascular strands in the primordia.

### *Flux-Based* Polarization Model

The model is essentially based on the *flux-based* polarization hypothesis derived from the *canalization* concept, introduced by Sachs [14] who suggested that auxin transport is increased during the vascular induction by the auxin flux itself, leading to the canalization of the flux (for earlier mathematical formalizations see also [15]–[18],[37]. The model is inspired by the original Mitchison model revisited by Rolland-Lagan and Prusinkiewicz [16],[18].

#### Conservation law for the transport of auxin

We denote *a_i_* (mol·m^−3^) the concentration of auxin in a cell *i* and *p_i_*_,*n*_ (mol·m^−2^) the concentration of PIN proteins in the membrane facilitating transport from cell *i* to cell *n*. *V_i_* (m^3^) denotes cell volume and *N_i_* denotes the set of neighbouring cells of cell *i*. If *i* and *n* are two neighbouring cells, then *S_i_*_,*n*_ (m^2^) denotes the exchange surface between these two cells. We assume that the auxin variation rate results from the combination of three processes: (i) diffusion, (ii) active transport of auxin by PIN, and (iii) local cell auxin synthesis and decay (see Auxin Hypotheses 1–4).
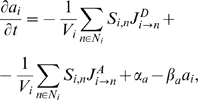
(1)where 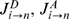 are the fluxes of auxin (mol·m^−2^·s^−1^) due to diffusion from cell *i* to its neighbouring cell *n*, active transport from cell *i* to *n* respectively. By convention, out-going fluxes are positive, incoming fluxes are negative. *α_a_* (mol·m^−3^·s^−1^) is a constant that describes the rate at which auxin is produced in cells and *β_a_* (s^−1^) defines the rate of auxin decay. Diffusion is modelled using Fick's First Law, 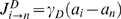 where *γ_D_* is the constant of permeability reflecting the capability of auxin to move across the membrane (m·s^−1^). In his original paper from 1981, Mitchison proposed to model the flux due to active transport across a *membrane* between cells *i* and *n* as 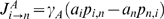 where *γ_A_* (m^3^·mol^−1^·s^−1^) characterizes the transport efficiency of the PIN pumps. Hence the auxin variation rate in a cell *i* can be expressed as:
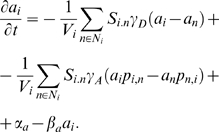
(2)


#### *Flux-based* polarization

According to Sachs' original concept, *canalization* relies on a feedback mechanism from the auxin fluxes on its transporters. More precisely, we assume that the concentration of PIN proteins *p_i_*_,*n*_ in cell *i* transporting auxin to cell *n* changes due to (i) insertion in the *membrane* induced by the flux and (ii) background insertion and removal of PIN from the *membrane*. The net flux of auxin that crosses *membrane* from cell *i* to cell *n* is 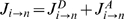.
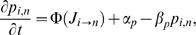
(3)where Φ defines the intensity of PIN insertion into the *membrane* due to the feedback of the auxin flux, *α_p_* (mol·m^−2^·s^−1^) describes the rate of background PIN insertion into the *membrane*, and *β_p_* (s^−1^) the background removal rate from the *membrane*. Depending on the nature of the Φ function, different types of canalization regimes can be obtained ([17] and see below). In this paper we use two types of functions: a linear function Φ*_L_*(*J_i→n_*) = *κ*(*J_i→n_*/*J*_ref_) and a quadratic function Φ*_Q_*(*J_i→n_*) = *κ*(*J_i→n_*/*J*_ref_)^2^, where *κ* (mol·m^−2^·s^−1^) is a constant parameter and *J*_ref_ (mol·m^−2^·s^−1^) is an arbitrary reference flux that makes it possible to keep constant units in the equation. For a negative net flux the Φ functions are truncated to 0, which means that no additional PIN is inserted in the *membranes* for which more auxin particles come in than particles come out.

### SAM Model

As mentioned above we suppose that auxin flows essentially in two separated conduits: the L1 layer and the subepidermal layers. The two systems meet at the primordia cells. This very localized coupling between epidermal and subepidermal domains makes it possible to model the transport in each pathway independently and to account for their interaction at the sites of primordia only.

#### Epidermal model

We represent the L1 layer by a set of polygonal cells forming a 2D surface. Similarly to other models of auxin transport at the SAM [24],[25], the inter-cellular space was not modelled as a compartment of its own (however see [38]) and the contact between cells was abstracted as a single separation (referred to as *membrane*) allowing auxin molecules to flow between adjacent cells and PIN molecules to accumulate on either side. To model phyllotaxis we included certain topological and geometrical assumptions. We identify a particular point *z* as the meristem centre. Different zones of the meristem are defined relatively to this centre *z*. The centroid of each cell *i* is denoted by *o_i_*. The central zone, 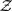, is the set of cells whose centroids have a Euclidean distance to the meristem centre *z* less than or equal to the constant radius 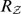. Similarly, a cell *i* belongs to the peripheral (or competence) zone 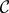 when the distance between its centroid *o_i_* and the meristem centre *z* is less than or equal to 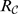 and greater than 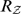. Cells *i* in the peripheral zone can be promoted to primordia cells (which is denoted by *i* ∈ 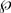).

#### Subepidermal model

Second, to model the vascular pathways below the primordia, we designed a 2D model of a longitudinal section of the SAM where the connection between the epidermal and subepidermal layers could be explicitly represented. In the subepidermal layer, the definition of the zones 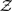, 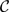, and of primordia cells 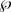 is analogous to that of the epidermal model. Cells are also represented as 2D planar polygons whose edges represent cell *membranes*.

#### Growth of the SAM

To simulate meristem dynamics, we used a purely kinetic description of meristem growth [25]. We explicitly defined the velocity *v* of every point at the meristem surface in a reference frame attached to the meristem centre *z*. The velocity *v*(*x*) of a point *x* at the meristem surface is assumed to be proportional to its distance to the meristem centre: *v*(*x*) = *ρ*|*x*−*z*|, thus simulating isotropic radial growth [2]. The constant *ρ* defines the relative elementary growth rate in the radial direction [39]. Due to this global kinetic process, the vertices of each cell move toward the meristem periphery with a velocity growing exponentially. This makes the cells grow smoothly as they move away from the meristem centre. As soon as a cell surface exceeds a constant threshold *S*_max_, the cell divides by creating a new wall inside. The position of this wall is computed using a modification of the optimization criterion introduced by Nakielski [40], i.e., finding a wall that both minimizes the distance between two opposite walls of the cell and that divides the cell into two polygons with the same surface. Then, similarly to [25], the cell vertices of newly created walls are slightly moved toward each other to provide a more realistic aspect. After a cell division event, the auxin concentration and PIN concentration in the *membranes* are inherited by the daughter cells from their parent. Primordium identity is inherited by randomly choosing one daughter of the primordium cell as the new primordium cell. The new *membrane* is initialized with *α_p_*/*β_p_* concentration of PIN on both sides. Finally, to keep a constant size of the overall simulation, a cell *i* that is too far away from the meristem centre *z* (its centroid *o_i_* is at a distance greater than *R*_sim_) is removed from the simulation.

In order to integrate in a single model the different processes involved in the system, i.e., auxin transport, cell growth, division, PIN allocation, and cell differentiation, we assume that these processes take place at different scales. Auxin transport is supposed to be much faster than growth and cell differentiation so that in the growing meristem, auxin concentrations are always at equilibrium.

### Practical Aspects of Simulation

#### Numerical solving

The non-linear system of equations describing the *flux-based* polarization model is integrated using the Scipy package designed for ODE solving [41]. The integration algorithm uses the Adams predictor-corrector method in the non-stiff case [42]. Solver iterations are performed until a stable state is obtained, i.e., until change in auxin concentration becomes less than a predefined threshold value *ε*_min_ in every cell.

#### Boundary and initial conditions

The boundary conditions for every simulation are specified in the supplementary material (Text S1). In most simulations boundary cells do not receive any auxin flux from the outside and we assume fixed, null concentration in sinks. In all simulations we assume that the initial auxin concentrations are null and PIN concentration on both sides of the *membrane* are initiated with a basic amount of PIN *α_p_*/*β_p_*.

#### Visualisation and simulation environment

The visualization of tissue simulations was carried out with PlantGL, an open-source graphic toolkit for the creation, simulation and analysis of 3D virtual plants [43] available in the OpenAlea software platform for plant modelling [44].

#### General convention for figures

In all figures representing 1D or 2D tissues, we adopted the following graphical conventions: the absence of auxin in a cell is represented by black while the highest concentration is shown in bright green. Intermediate concentrations are represented by interpolations between these two extremes (see Text S1). PIN transporters at the *membrane* of a cell *i* are represented as a red line. The thickness of this line is proportional to the amount of PIN.

#### Supplementary materials

For every figure showing a dynamic system, we provide a corresponding movie to capture system dynamics (Videos S1, S2, S3, S4, S5, S6, S7, S8, S9, S10, S11, S12, S13, S14, S15, S16 and S17). Movies are available as supplementary materials and named after the figures. Supplementary text is provided, specifying equations, parameters, boundary and initial conditions and display specific conventions (Text S1).

## Results

The study of systems controlled by *flux-based* polarization is not straightforward as the process relies on a feedback loop between auxin concentrations and auxin fluxes in tissues. To address this problem, we first defined different remarkable properties of the *flux-based* polarization that are essential in the generation of patterns. These properties are illustrated on simplified 1D or 2D “virtual tissues”. The sensitivity of the model for different parameters was tested. As expected, the sytem was more dependent on certain parameter values, but overall the results were robust (Text S1). Based on this analysis, we then investigated the ability of *flux-based* polarization to produce phyllotactic patterns at the SAM in a way that is consistent with the current biological knowledge and observations.

### *Flux-Based* Polarization Amplifies Fluxes

**Figure 2:**
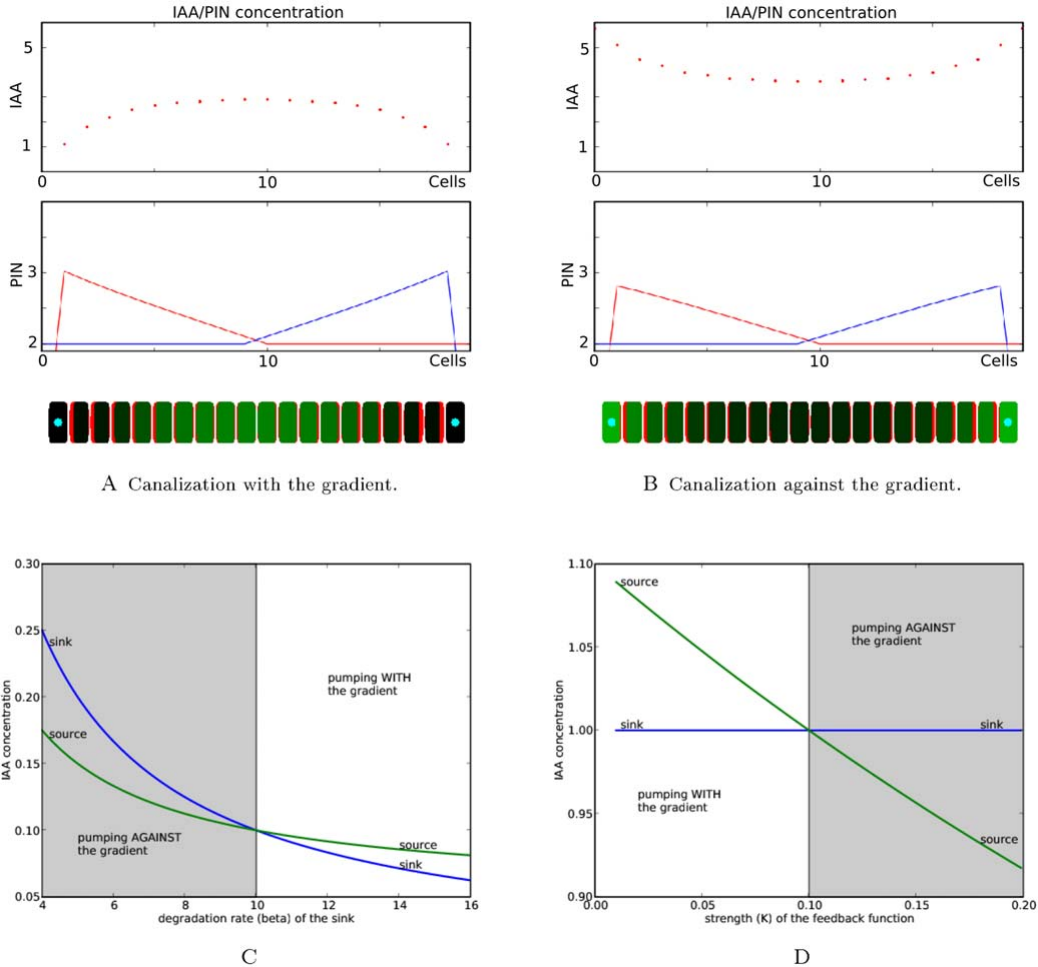
Canalization in a 1-dimensional cellular system. (A,B) show the system consists of 20 linearly aligned cells. Boundary cells are acting as sinks; hence they evacuate or degrade auxin. At the start of the simulation the cells do not contain auxin. Then the simulation runs until a stable state emerges. On the two first plots (A,B) the blue and red lines correspond to PIN concentrations at the right and left side of the membranes respectively. The two systems differ only by the way auxin is removed: in (A) we assume that the removal in the sink cells is very efficient whereas in (B) the removal efficiency is limited. This difference leads us to two different stationary patterns in which the auxin gradients are opposite and the sink cells are minima of auxin (A) or maxima (B). (C,D) present a further analysis of the conditions leading to pumping against or with the gradient. For this purpose a system of two cells sharing one membrane was analysed. (C) presents the concentrations of auxin in the two cells in the stable state as a function of the degradation rate *β* of the sink. The green curve corresponds to auxin concentrations in the source cell, the blue curve corresponds to auxin concentrations in the sink cell. In the grey region, pumping is carried out against the auxin gradient, while in the white region, pumping follows the gradient. (D) shows similar curves for the variation of the feedback strength *κ* of flux on PIN synthesis.

The *flux-based* hypothesis, proposes that any small flux between two cells in the system will reinforce itself by increasing the local amount of PIN, thus initiating a positive feedback loop. Initial fluxes may typically be generated by diffusion between zones with different concentrations. We illustrated this phenomenon on a 1-dimensional tissue with two perfect auxin sinks at both extremities (Figure 2A). Auxin is produced in every cell except the sink cells. Initially, the highest flux appears close to the sink cells, due to diffusion. This small initial flux is subsequently reinforced by a polar allocation of PIN transporters favoring the evacuation of auxin in the direction initiated by the original flux. If the auxin sink is maintained, the auxin flux reaches a stable state with the maximum concentration of auxin appearing at the maximal distance from both sinks (Figure 2A). This figure also shows that the concentration of PIN at each cell *membrane* linearly increases from the location of the auxin maximum up to the sinks. This is because each cell is producing auxin at a constant rate *α_a_* and in the stationary state the amount of removed auxin must be balanced by auxin synthesis (if we neglect auxin degradation). It implies that the auxin flux should grow linearly in the direction of the closest sink. If the feedback function Φ is linear, this results in a linearly increasing allocation of PINs to the cell *membranes* in the direction of the closest sink.

### *Flux-Based* Polarization Allows Auxin To Flow with or against Auxin Gradients

Although the molecular mechanism underlying PIN polarization is still unknown, PIN proteins can polarize either *with* or *against* the gradient of auxin [8],[22],[45]. If a unique transport mechanism is operating in the plant it should thus be able to reproduce this property. In the previous example, auxin fluxes were amplified from regions of high concentration of auxin to regions of low concentration (Figure 2A). Auxin thus flowed *with* the auxin gradient.

To show that *flux-based* polarization can also lead to flow against the gradient, we modified the above 1-dimensional model by weakening the sinks in such a way that they were only able to degrade auxin at a finite constant rate. This simple modification produces a drastic change in the system's behaviour. The auxin gradient is now reversed in the stable state, with highest concentrations at the sink locations and lowest in the places *maximizing* the distance to all sinks (Figure 2B).

To study the conditions for either pumping *with* or *against* the gradient, we considered a system of two cells sharing a *membrane*. One cell is a source of auxin while the other acts as a sink destroying auxin at a constant rate. Once this system reaches a stable state, the net flux across the *membranes* separating the two cells is exactly equal to the rate at which the source creates auxin and leads to a polarization of PIN from the source to the sink. Depending on the model parameters, the system can reach different levels of concentration in both cells. Two regimes may be obtained as shown by the graphs (Figure 2C and 2D). The transition between both regimes, pumping *with* or *against* the gradient, can be obtained by varying different parameters of the model such as the feedback strength and the degradation rate (2).

### *Flux-Based* Polarization Has Two Different Regimes (*Weak* and *Strong*)

**Figure 3:**
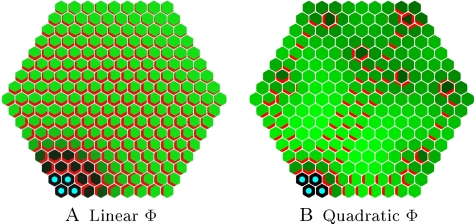
Weak and strong regimes on 2D hexagonal lattices. The sink cells are tagged with blue dots. (A) shows the stable state in case of a weak regime. At the end of the simulation the auxin concentration is progressively increasing with the distance from the sink. PIN, marked in red, is present in all cells leading to a laminar flow over the entire surface. (B) shows the stable state of a strong regime with one sink leading to the formation of canals (where PIN is present) and patches of cells without transporter. This system corresponds to the original *canalization* concept.

Initially, the *flux-based* polarization hypothesis was introduced to model the formation of vascular canals in stem and leaf tissues, as an integral part of the *canalization* concept [15]–[17],[19],[37]. Using this mechanism in the meristem may seem in contradiction with the absence of canals at the meristem surface. Feugier et al. [17] demonstrated that a *fluxed-based* polarization mechanism where the feedback function Φ was linear did not result in the formation of canals in a tissue. We further confirmed this by comparing the behaviour of auxin transporters in a 2D sheet of cells showing weak or strong feedback. When the feedback function Φ is non-accelerating (increasing linearly or less rapidly than linearly) the process creates laminar flows transported by homogeneous arrangement of PINs and converging to the sink (Figure 3A). We refer to such a system as a *weak* regime. Conversely, when the feedback function Φ is *accelerating* (increasing more rapidly than linearly), canals appear, creating branching structures in the 2D tissue (Figure 3B). We will call such a system a *strong* regime. In both cases, fluxes may be oriented with or against the gradient, depending on the model parameters and boundary conditions.

### The *Weak* Regime Can Produce Fields of Lateral Inhibition of Varying Intensities

As explained earlier, the most widely accepted theory of phyllotaxy relies on the formation of inhibitory fields around each primordium. Recent models propose that these fields are in fact zones where auxin is depleted [2],[3],[6]. To show that *flux-based* polarization can indeed be considered as a plausible mechanism, we demonstrate that it can generate such inhibitory fields with varying intensities.

**Figure 4:**
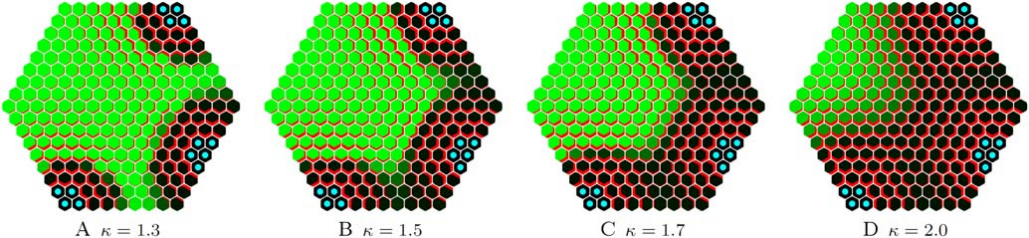
Inhibitory fields induced by a flux-based polarization system. The size of the field changes according to the value of parameter *κ* which regulates the feedback of fluxes on PIN pumps synthesis.

A *weak* regime (as in Figure 3A) leads to the formation of a zone around the sink where auxin is depleted. The intensity of the auxin depletion fields around sinks can be changed by tuning the parameter *κ* that controls the feedback level of auxin fluxes on PIN insertion in the *membranes*. Figure 4 shows the extent of inhibitory fields (in black) around the blue sinks for increasing values of parameter *κ*. PIN is regularly distributed throughout the tissue, with a polarity that is determined by the relative distance of the cell to the different sinks. The weak regime thus makes it possible to vary the auxin depletion level around sinks. It therefore provides a plausible explanation for the formation of inhibitory fields during organ initiation at the SAM.

### *Flux-Based* Polarization as a Source of Patterning in a Growing Structure

**Figure 5:**
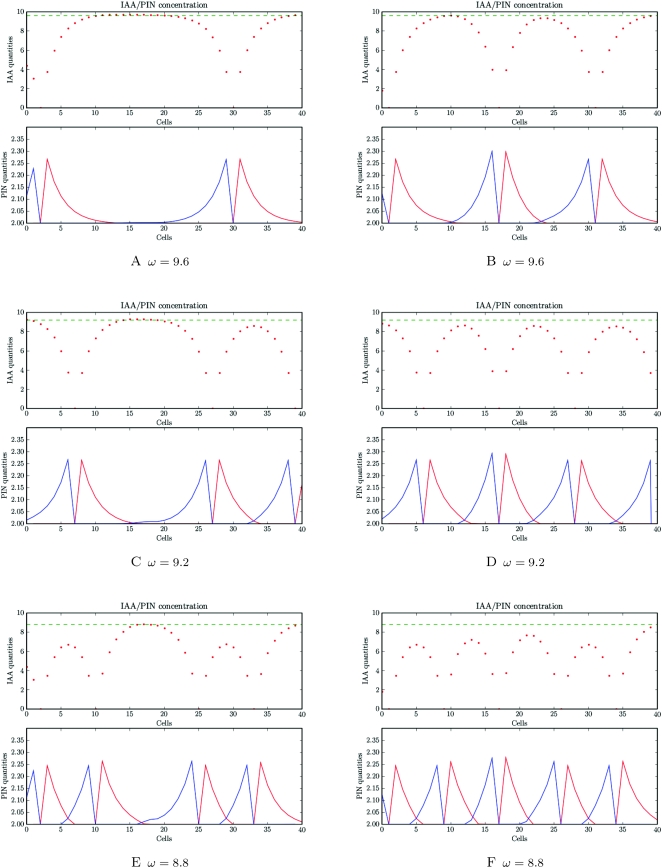
Dynamic patterning with flux-based polarization. (A,B), (C,D), and (E,F) present three simulations with different thresholds *ω* for primordium initiation. (A,C,E) present the step just before primordium initiation and (B,D,F) present the step just after primordium initiation. The frequency of primordium initiation increases with a decrease of the initiation threshold *ω*.

The mechanism that controls PIN orientation in cells takes place in a growing structure. Therefore we constructed a dynamic model with dividing and growing cells. As before, we assume that all cells create auxin except for a limited number of cells marked as sinks in which auxin concentration is fixed at 0. To produce phyllotactic patterns, the combination of *flux-based* polarization hypothesis and tissue growth should therefore show a recurrent, temporal patterning property. We show this property in a simplified 1D model by introducing a sink creation threshold, i.e., an auxin concentration at which a new auxin sink is created. In a growing system, neighbouring auxin sinks are pushed apart. Due to the weakening of the sink influence and the constant local hormone production the level of auxin increases in the zone between these two sinks. At a particular auxin threshold (the sink initiation threshold *ω*), the amount of hormone is sufficient to initiate a new sink at the location which is the farthest from the two sinks (Figure 5A and 5B). As a result of sink creation, some of the PIN pumps reverse toward the new sink, with PIN and auxin patterns similar to that of the previous sinks. By changing the sink initiation threshold *ω*, it is possible to augment or to decrease the initiation frequency (Figure 5C–F). In the supplementary materials we show in details how the initiation frequency depends on different model parameters (Text S1).

### *Flux-Based* Polarization Model Can Reproduce Observed PIN Maps and Realistic Influence Zones

**Figure 6:**
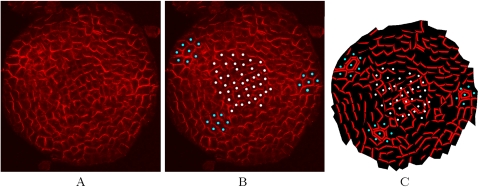
SAM digitalization. (A) shows the PIN distribution in a real meristem obtained using immunolabelling (top view). (B) shows the same image with marked primordia cells (blue dots) and central zone cells (white dots). (C) shows the reproduction of PIN distribution and polarity in a digitized version of the same image.

In Barbier et al. (2006), we showed that the distribution of PIN at the SAM (called “PIN map”) has a number of specific features [10]. As illustrated in Figure 6, PIN labelled membranes are pointing towards their nearest primordium (blue dots in Figures 6B and 6C). In addition, a significant number of cells appear to transport auxin to the meristem summit. A plausible model of phyllotaxy should be able to reproduce similar distributions of PIN.

To determine to what extent the *flux-based* polarization model could reproduce realistic PIN maps, we digitized the cell walls on the images of real meristems, immunolabelled to visualize PIN. We recorded the PIN orientation in each cell as described in [10] (called *real PIN maps* as in Figure 6C). The position of each primordium could be clearly identified as indicated by the convergence of PIN toward particular cells and the presence of vascular strands below these primordia seen on other sections of the same meristem (blue dots in Figure 6B, longitudinal image data not shown). We also designated a central zone of about 6 cells in diameter at the meristem summit. This zone is usually free of primordia in the wild type *Arabidopsis* SAM.

**Figure 7:**
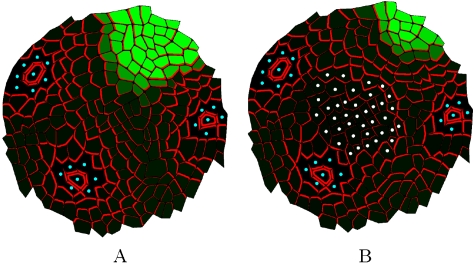
Simulation of auxin transport in a digitized meristem based on the flux-based polarization hypothesis. The cells and primordia of the real meristem shown in Figure 6 were used to initialize the system, and the virtual PIN maps were then calculated based on the flux-based polarization hypothesis. Green intensity is proportional to the virtual auxin concentration. (A) shows a simulation where the centre plays no special role in the auxin flux. (B) shows a simulation where the centre degrades auxin.

We then simulated the emerging arrangement of PIN distributions according to the *flux-based* polarization hypothesis on the digitized maps. Primordia were considered as perfect sinks while all other cells in the meristem were assumed to produce auxin at a fixed rate according to Equation 2. The resulting PIN distributions are shown in Figure 7. Close to the primordia, the simulated PIN arrangements are converging towards the sink cell and look similar to the PIN arrangements on the real PIN maps (Figure 6A). Besides, auxin accumulates at the position where one would expect the next initium in a spiral phyllotaxy (Figure 7A). However, contrary to real PIN maps, virtual PIN patterns did not show any significant converging tendency towards the centre of the meristem. To overcome this discrepancy, we made a second simulation, where the cells in the meristem centre were assumed to degrade auxin at a higher rate. While the convergence of PIN toward the primordia cells is preserved, an additional convergence of PIN toward the centre is now observed, reflecting more faithfully the observed distributions of PIN in the immunolabelling images (compare Figures 6C and 7B). The same result were obtained by reducing the synthesis of auxin in the central zone of the meristem (result not shown).

**Figure 8:**
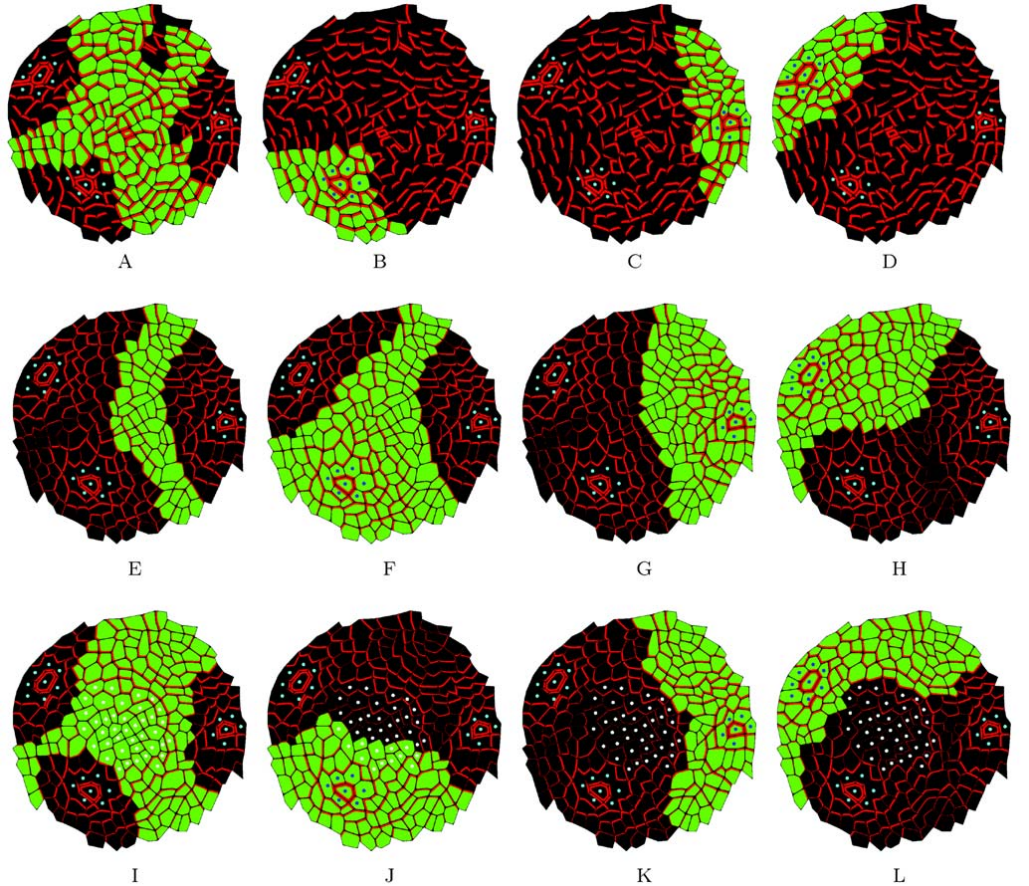
Influence zone analysis. The influence zones calculated for the central zone (first column), *P*_0_ (second column), *P*_1_ (third column), *P*_2_ (fourth column) in real and simulated maps. (A–D) show the influence zones in a real map. (E–H) show the influence zones in digitized maps where PIN labelling was calculated based on the flux-based hypothesis. In this simulation, cells in the central zone are identical to other cells. Primordia (blue cells) are perfect sinks. (I–L) shows a simulation based on the flux-based polarization hypothesis, but here both primordia and cells in the central zone (white cells) are removing auxin. Note that qualitatively the last simulation shows a better match with the original real map.

To go beyond a simple visual inspection for similarity, we computed the so-called *influence zones* of the primordia and of the central zone in the real meristem and compared them to those in the simulated meristems. The influence zone of a region (i.e., a cluster of cells) is the set of meristem cells that are connected to a cell of the considered region through a path of PIN arcs oriented in the direction of this region. Figure 8 shows the influence zones of different regions (centre and primordia) on real (Figure 8A–D) and simulated (Figure 8E–L) PIN maps. In real maps, pumps are distributed in such a way that auxin can reach the central zone from all the directions between each pair of primordia (with a small auxin pathway between primordia *P*_0_ and *P*_2_, a larger one between primordia *P*_0_ and *P*_1_ and the largest pathway between *P*_1_ and *P*_2_). Influence zones of the primordia are restricted to the neighbourhood of each primordium and do not extensively overlap with the cells of the central zone. We then computed the influence zones from the first simulation where the central zone did not act as a sink. This showed important differences with the real maps. For the central zone , only two auxin pathways of equivalent width could be observed while the pathway between *P*_0_ and *P*_2_ had disappeared (Figure 8E). In addition, the influence zone of *P*_0_ largely crossed the meristem centre in 8F in contrast to what was observed on the real map. The influence zones of the simulations with auxin depletion in the centre showed better agreement with the influence zones computed from real PIN maps: with three auxin pathways of gradually increasing width going to the meristem centre and the influence zones of primordia being almost non-overlapping with cells in the central zone (Figure 8I–L).

### Formation of Phyllotactic Patterns and Provascular Strands

Based on the preceding results, we designed a dynamic model of phyllotaxy using the *flux-based* polarization hypothesis. The epidermal and subepidermal layers were assumed to be relatively independent, except at the primordia were the two interact by exchanging auxin. This assumption was based on the generally accepted hypothesis that auxin is accumulated in the L1 layer due to the presence of influx carriers of the AUX/LAX family on the cell membranes [7]. This made it possible to simulate auxin transport at the surface and in internal layers separately and to summarize their interactions as boundary conditions. Since in the L1 layer no channels of auxin transport are observed, we supposed that the weak regime prevailed at the surface. For vein formation in inner tissues, we supposed that a strong regime was active.

**Figure 9:**
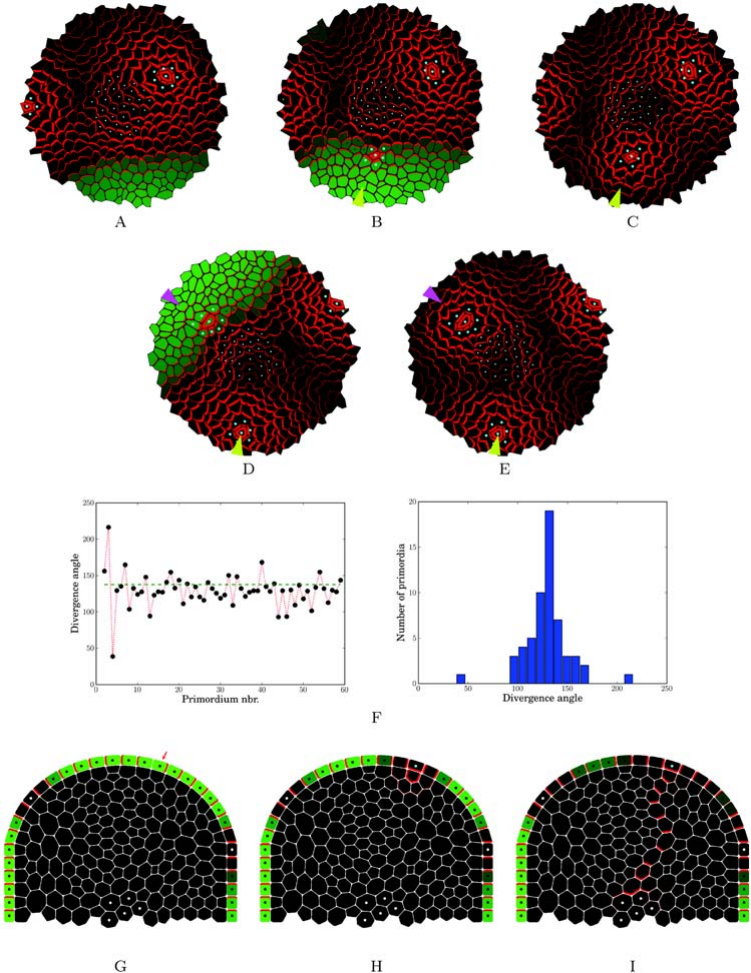
Dynamic models. (A–E) 2D top view of a virtual meristem following the *flux-based* model showing the dynamics of PIN distribution, auxin concentrations and primordia initiation. The sequence shows the initiation of two consecutive primordia (arrowheads). The sequence starts with auxin accumulation in the zone that is the farthest away from the existing primordia. When a threshold is reached, the maximum acquires primordium identity and becomes a sink. (F) shows the variation of the angles between 65 consecutive primordia. Note that the mean value is close to the golden angle (137°5) typical for spiralled phyllotaxis as observed in *Arabidopsis*. (G–I) 2D transversal cut of a virtual meristem following the *flux-based* model showing the dynamics of PIN distribution, leading to the formation of a provascular strand of cells that transport auxin downwards. Detailed description in the text.

The simulations using the model characteristics described above resulted in a dynamic pattern of auxin distribution and primordium formation. The following general scenario was observed. In the L1 layer, each primordium evacuates auxin by its provascular system to the inner parts of the meristem. In the L1, the primordium can thus be considered as a sink depleting auxin in its immediate neighborhood. This in turn inhibits the formation of new primordia close to the existing ones (Figure 9A). Due to cell growth, primordia progressively move away from each other, which allows the accumulation of auxin in cells sufficiently distant from these young organs. As a result a new maximum of auxin concentration gradually appears in the region maximally separated from all primordia, thus defining the location of the next initium (Figure 9G). As soon as the auxin concentration exceeds a predefined threshold in a cell belonging to the competent zone surrounding the central zone, this cell and its immediate neighbours acquire primordium identity (Figure 9B). This implies that auxin can leak at the initium location into the inner layers, which triggers the creation of the primordium vascular strand (Figure 9H). The vein being formed below the initium drains the auxin out from the L1 layer and converts the initium into an auxin minimum (Figure 9C and 9H). The flux induced by this process reverses pump polarizations in the direction of the initium in the L1 (Figure 9C and 9I). Then, due to tissue growth, new space becomes available allowing the system to generate a new initium (Figure 9D and 9E). This system is able to produce a stable phyllotactic pattern, with a mean angle close to the golden angle, 137°5 (Figure 9F) observed in *Arabidopsis* and characteristic for spiralled phyllotaxis.

An obvious simplication in this model is the assumption that an instant drop in the auxin concentration occurs when cells of L1 acquire primordium identity. However, modifying the rate at which the auxin concentration changes at the initium showed that an immediate drop gives qualitatively the same patterns as a gradual reduction over time (simulation not included). Since this would add another parameter to the model we kept this simplification.

## Discussion

We describe here a *flux-based* model, that provides a realistic explanation for phyllotaxis, predicting patterns of PIN distribution that are very similar to the observed ones. The model leads to a classical inhibitory field scenario where the very young primordia pumps auxin towards the inner tissues, draining the hormone away from their immediate vicinity. As long as these sinks are close to the competence zone, no new primordia can be formed. However, as growth drives the sinks away, auxin concentration can build up again locally because of synthesis and transport, creating a new auxin maximum. The model proposes that the import capacity of the L1 layer at the surface is overridden when a certain auxin concentration threshold is reached after which the hormone starts to leak away to inner tissues. This initial diffusion-driven flux will be reinforced by *flux-based* polarization. This in turn will rapidly create an auxin transport channel connecting the local surface maximum to the inner vasculature and transforming it into a sink. The main requirement here is that *flux-based* polarization should be relatively weak at the surface, switching to a strong regime in inwards directed fluxes. This switch from one regime to the other could directly depend on the amount of auxin flowing through the cell, but it could also be activated indirectly as part of the differentiation process induced by high auxin concentrations. In such a scenario even a small leakage from the surface to the inner parts would be very rapidly amplified

### *Flux-Based* Polarization Hypothesis as a Unifying Concept

**Figure 10:**
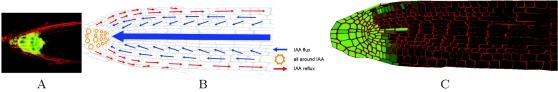
*Flux-based* polarization model of the root meristem. *Flux-based* polarization hypothesis is compatible with the maintenance of an auxin maximum and with the general organization of PIN at the root tip. (A) shows the existence of a stable auxin maximum at the root tip as evidenced by the DR5:GFP marker (Ottenschlager 2003; Grieneisen 2007). At the shoot apex, the general organization of the different PIN transporters in the different tissues suggests a flux going downward via the vascular tissues and than spreading out over superficial layers “like a fountain” (B). (C) shows a digitized root apex based on a real optical section from an *Arabidopsis* root (not shown, image taken by Tom Beeckman). Similarly to PIN maps at the shoot apex, the polarity of PIN was recorded in each cell. This PIN map was used as an initial condition for the simulation. The cellular system was provided with a fixed global quantity of auxin initially divided equally over the tissue. In addition, two border cells of the epidermis were chosen as auxin sinks, to comply with the biological assumption that a fraction of auxin is evacuated from the root tip along the epidermis (Swarup 2005). Auxin arriving in these sink cells is completely depleted at each simulation step. In addition, a permanent auxin source was added on the border of the central vascular system providing auxin in a constant fashion, in accordance to biological auxin source localization in the vascular bundle. Simulations revealed that transporter dynamics based on *flux-based* polarization are sufficient to enable and maintain auxin accumulation in the collumella and quiescent centre (C), as observed in (A). Additionally, realistic transporter distribution profile was maintained by the *flux-based* polarization mechanism.

*Flux-based* polarization provides an alternative explanation for phyllotaxis. Indeed, since it allows for transport with and against gradients, it also provides a plausible explanation for the stable auxin maxima observed in roots and leaves. This is illustrated in Figure 10 where we have reproduced PIN and auxin distributions in the root meristem using the flux based hypothesis. An important caveat is that two different regimes are required in the model. As shown previously by Feugier et al., only the weak regime can explain the absence of canals as observed at the meristem surface [17]. In this context it is important to note that the weak regime has some characteristics in common with diffusion. In particular, both processes can lead to auxin transport down the gradient. However, as auxin cannot freely diffuse between the cells, the weak regime is fundamentally different and can only function if the cell is able to sense fluxes. As long as the precise mechanism of PIN localisation is not known, it will be difficult to predict whether each regime would require a completely different cellular mechanism which would go against the idea of a unifying concept. However, it seems reasonable to propose that both regimes correspond to different states of the same flux sensing mechanism. We therefore conclude that the *flux-based* hypothesis remains a potential unifying mechanism for auxin transport throughout the plant.

### Confronting the *Flux-Based* Polarization Hypotheses with Experimental Results

Having established that the *flux-based* polarization model can reproduce phyllotactic patterns, it now becomes important to test the hypothesis as rigorously as possible. We have made a first step towards this procedure by comparing the predicted PIN protein patterns with the observed ones. While this in itself is a stringent test, so far not performed on other models, the *flux-based* model should also be coherent with other existing data. In the following paragraphs we will discuss a number of its implications.

### Auxin Concentrations at the L1 Layer

Like the *concentration-based* model, the *flux-based* model requires that the patterning process mainly occurs in the L1 layer. This is based on the idea that auxin is concentrated there by auxin importers (AUX1 and LAX proteins) that are strongly expressed in the L1. In addition, the highly organized distribution of PIN at the meristem surface also indicates that the patterning process mainly occurs in the L1 layer. This might seem contradictory with the phenotypes of mutants where the auxin importers are impaired and which are still able to generate primordia. It should be noted, however, that other factors such as the human multiple drug resistance/P-glycoproteins (MDR/PGPs) are also involved in auxin influx [46] and could guarantee the presence of sufficiently high levels of auxin in the L1 layer when the AUX/LAX proteins are inactivated. We therefore conclude that overall our model is in line with the experimental data supporting a major role of the meristem surface in phyllotaxis.

### Auxin and the Central Zone

An intriguing aspect of our model concerns the central zone (CZ) cells. Like the *concentration-based* model, the *flux-based* model does not require any particular property of this zone, other than a lack of competence to generate a primordium. We could, however, only obtain realistic patterns of PIN distribution when we attributed a sink function to this zone. This is in line with earlier observations by Barbier et al. (2006) who provided evidence that the CZ receives auxin fluxes [10]. However, the sink function of the CZ required by the *flux-based* model seems in contradiction with the same study showing the presence of relatively high auxin levels there. It is therefore important to note that in the *flux-based* model the concentration in the CZ is not much lower than in the other peripheral zone cells, and is certainly significantly higher than in the primordium cells. Therefore on average the model would predict higher concentrations in the CZ than in the periphery as reported by Barbier et al. (2006). As a result there does not appear to be any conflict between earlier observations and the *flux-based* hypothesis.

### Auxin Concentrations and PIN Dynamics

The *flux-based* model scenario proposes that a relatively broad auxin peak leads to an inward flow which rapidly leads to the formation of an auxin sink. It is the formation of this sink that will reorient the PIN transporters in surrounding cells. This scenario seems in contradiction with earlier observations suggesting that (i) the reorientation of PIN transporters precedes the initiation of the young organ and (ii) that an auxin maximum is maintained at the young primordium. There are several explanation for these apparent contradictions.

**Figure 11:**
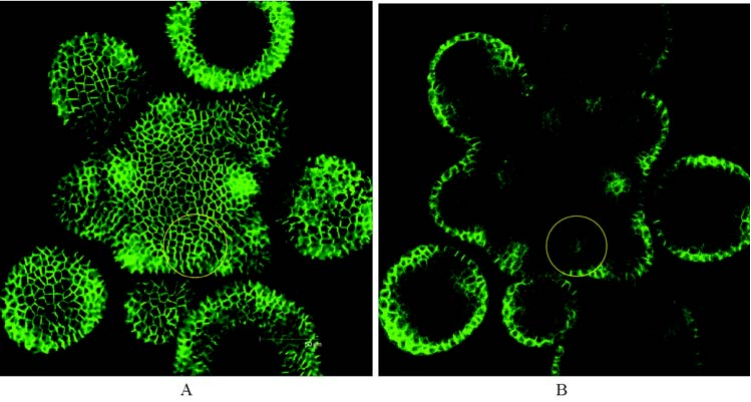
Confocal image of a living meristem expressing a fluorescent version of PIN (PIN1:GFP). (A) shows the higher expression of PIN at the surface around new primordia is clearly observed. The yellow circle indicates the site where the next organ will be initiated. The images show no very obvious reorientation of PIN orientation at this site, although a provascular strand expressing PIN is clearly observed at this site (yellow circle on the individual section of the same meristem in (B)).

First, it has not been unambiguously established that the PIN transporters orient before the initiation of the inward flux marked by the formation of the provascular strand. Heisler et al. (2005) showed that there is some reorganisation of PINs before this strand is formed but did not report clear converging transporters [47]. In accordance with this, we have observed the presence of PIN in the internal tissues at a moment where the transporters were not clearly converging (Figure 11). We therefore conclude that the precise timing has not been sufficiently well established to draw clear conclusions.

**Figure 12:**
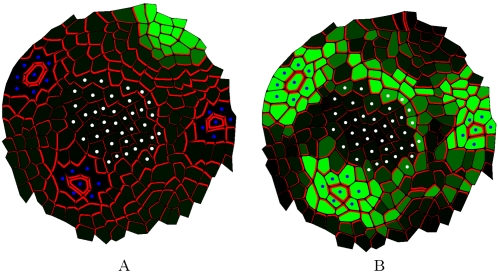
The simulation 7B results showing the predicted levels of auxin concentration (A) and flux intensity (B). The simulation 7B results showing the predicted levels of auxin concentration (A) and flux intensity (B). Note the sharp peaks observed in (B), comparable to DR5rev:GFP peaks observed in vivo [10].

Second, the supposed stable auxin maximum at the young initium has been revealed using the so-called DR5rev promoter driving the green fluorescent protein (GFP). This promoter contains an auxin responsive element and is activated by auxin responsive transcription factors. While it is often presented as a quantitative auxin sensor, it is in fact only a very indirect marker which not only depends on the amount of hormone but also on the capacity of cells to react to it. In addition the fluorescent GFP marker can be stable for prolonged periods, and could therefore mask rapid changes in the activity of the promoter. It is therefore possible that the precise fluctuations in auxin concentrations at the primordium have not been unambiguously established. In addition, DR5rev might not only mark high auxin concentrations but could also be activated by other factors For instance, high levels of another hormone, brassinolide, can activate DR5 in the apparent absence of changes in auxin levels [48]. At this stage, we even cannot exclude that DR5rev reacts to auxin fluxes, although this remains speculative. Interestingly, our simulations show that a *flux-based* activation of DR5rev would give sharp maxima, very comparable to what is observed in vivo (Figure 12).

### General Conclusion

In this study we have shown that the *flux-based* polarization model is a plausible alternative to the existing *concentration-based* model for phyllotaxis. Further experimentation is now required to distinguish between the two models. A careful and quantified description of cell behaviour (e.g., PIN distribution) should be part of this approach. We are further testing this by using transgenic approaches aimed at modifying the capacity of these cells to transport auxin or by changing the auxin content in the same cells. A major scientific question concerns the actual mechanism involved in auxin transport. Indeed a better insight into the process might also help to validate one or the other polarization hypothesis. It is important to note, that both the *flux-based* and *concentration- based* models are obvious abstractions of reality. They both do not, for instance, take into account inter-cellular spaces nor do they indicate how auxin fluxes or auxin concentration gradients are sensed. A process like the *flux-based* polarization mechanism described here could, therefore, be much more complex than just PIN proteins sensing auxin particles flowing through the cell. What is important here is that the overall behaviour of the system can be described accurately by flux-based polarization model with predicted, testable properties.

## Supporting Information

Text S1 Equations, parameters, boundary and initial conditions, and display conventions. This file describes all the mathematical and practical details that have been used to perform the simulations used in the paper. In particular, all parameter values are provided for each experiment.
(0.31 MB PDF)

Video S1 Movie presents the dynamics of Figure 2A.
(0.31 MB AVI)

Video S2 Movie presents the dynamics of Figure 2B.
(0.37 MB AVI)

Video S3 Movie presents the dynamics of Figure 3A.
(1.00 MB AVI)

Video S4 Movie presents the dynamics of Figure 3B.
(2.43 MB AVI)

Video S5 Movie presents the dynamics of Figure 4A.
(0.97 MB AVI)

Video S6 Movie presents the dynamics of Figure 4B.
(0.95 MB AVI)

Video S7 Movie presents the dynamics of Figure 4C.
(0.95 MB AVI)

Video S8 Movie presents the dynamics of Figure 4D.
(0.80 MB AVI)

Video S9 Movie presents the dynamics of Figure 5A.
(4.15 MB AVI)

Video S10 Movie presents the dynamics of Figure 5C.
(4.24 MB AVI)

Video S11 Movie presents the dynamics of Figure 5E.
(4.17 MB AVI)

Video S12 Movie presents the dynamics of Figure 7A.
(1.45 MB AVI)

Video S13 Movie presents the dynamics of Figure 7B.
(0.74 MB AVI)

Video S14 Movie presents the dynamics of Figure 9A (part 1).
(8.96 MB AVI)

Video S15 Movie presents the dynamics of Figure 9A (part 2).
(7.38 MB AVI)

Video S16 Movie presents the dynamics of Figure 9G.
(0.92 MB AVI)

Video S17 Movie presents the dynamics of Figure 10A.
(1.66 MB AVI)
