# Supplementary material for: Flux-Based Transport Enhancement as a Plausible Unifying Mechanism for Auxin Transport in Meristem Development
Source: PLoS Comput Biol. 2008 Oct 31;4(10):e1000207. doi: 10.1371/journal.pcbi.1000207 (PMC2565506; doi:10.1371/journal.pcbi.1000207)
Supplement: Text S1 — Equations, parameters, boundary and initial conditions, and display conventions. This file describes all the mathematical and practical details that have been used to perform the simulations used in the paper. In particular, all parameter values are provided for each experiment. (0.31 MB PDF) [file pcbi.1000207.s001.pdf]

# Supplementary materials

## 1 Simulation overview

### 1.1 Conventions

**Notations:** In the equations describing changes in PIN and auxin concentrations we use the Iverson notation [1, 2]: if $\psi$ denotes a logic statement then,

$$[\psi] = \begin{cases} 1 & \text{if } \psi \text{ is True} \\ 0 & \text{otherwise} \end{cases}$$

**Supplementary movies:** For each figure demonstrating a system changing in time, a movie showing the process dynamics is provided. These movies are named after the Figures with supplementary letter "S" at the beginning (eg. the movie corresponding to Figure 2B is called "`VideoS2B.avi`").

**PIN display conventions:** The thickness of the line representing the PIN accumulation at a cell membrane is proportional to the computed PIN concentration at this membrane. However, high values are truncated to allow better inspection of visual results. The minimal displayed value of PIN concentration is always equal to $\alpha_p/\beta_p$, whereas the higher value corresponds to the maximum displayed concentration of PIN, which is $2\alpha_p/\beta_p$. All values of PIN exceeding this value are capped to $2\alpha_p/\beta_p$, allowing a ratio of 200% between extreme values.

**Auxin display conventions:** Auxin concentrations below minimal (resp. above maximal) threshold $a_{min}$ (resp. $a_{max}$) are depicted in black $RGB(0,0,0)$ (resp. in green $RGB(0,255,0)$). Intermediate values of auxin concentrations are depicted with a double linear interpolation function: a percentage $p_{mid}$ of the visible auxin range $[a_{max}, a_{min}]$ defines the auxin concentration $a_m$ for which the colour should be intermediate $RGB(0,127,0)$. Colours are then linearly interpolated between $a_{min}$ and $a_m$ and $a_m$ and $a_{max}$ respectively to render the colour of any auxin concentration within the interval $[a_{max}, a_{min}]$. Values for parameters $p_{mid}$, $a_{max}, a_{min}$ are defined for each simulation in table 1.

**Integration:** The number of snapshots was specified for each simulation, as well as fixed step $h$ which describes the time between each two consecutive time-points used for integration. For each simulation a number of steps is also given. This number is used to integrate the system with a given $h$ between taking a snapshot. For some simulations the number of steps may vary if the objective is to obtain a quasi stable state between the snapshots (eg. simulation of phyllotaxis). A stable state is reached when the change in $IAA$ concentrations in every cell becomes less than a predefined threshold value $\epsilon_{min}$ (using $L_\infty$ norm). In such a case the time interval between each snapshot may be different. If it is the case it is specified in the simulation details.

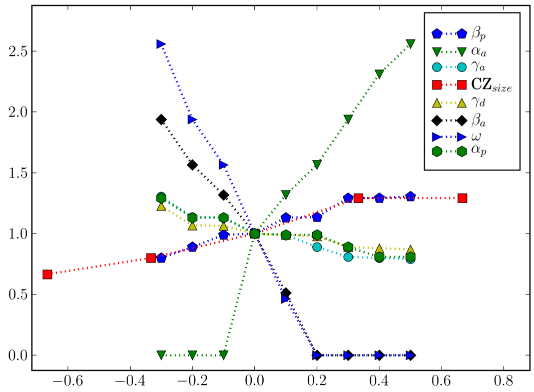


Fig. 1: Plot showing the influence of the parameter change on the relative change in frequency $f/f_9$ in a 1D model. On $x$-axis: $k$, on $y$-axis: $f/f_0$.

At each step a non-linear system of equations describing the flux-based polarization process is integrated using the SciPy package designed for ODE solving [3]. This package wraps ODE PACK, which is a collection of Fortran solvers for the initial value problem for Ordinary Differential Equation systems [4]. This solver allowed us to specify the precision on given integration interval (the step size is then adjusted automatically by solving algorithm).

## 1.2 Sensitivity analysis of the 1D flux-based polarization model

To test the sensitivity of the model results to variations of the model parameters, we made a sensitivity analysis on the 1D dynamic models of the type presented in Figure 5 in the main text and depicted in 5F simulation described below. We assume that the 1D medium is growing at constant velocity $v_0$ from the 1D tissue centre. Let us call $f$ the frequency at which a new primordium is generated during a particular simulation. $f$ typically depends on the model parameters: $\alpha_p, \beta_p, \alpha_a, \beta_a, \gamma_a.\gamma_d.\omega, CZ_{size}$. We define a reference frequency $f_0$ as the frequency corresponding to the model parameters defined in simulation 5F. To perform the analysis each parameter of the model is independently augmented by a factor $k\epsilon$ where $k \in [-3, -2, ..., 5]$ and $\epsilon = 10\%$. The competence zone size was also included in the tests, but due to its discrete nature it was augmented by a specific factor (see Figure 1 for the exact values). The Figure 1 shows the dependency of the relative change in frequency $f/f_o$ with respect to each parameter change.

The sensitivity analysis confirms a number of intuitive predictions:

1. The increase of auxin synthesis $\alpha_a$ (or decrease of auxin decay $\beta_a$) increases $f$. Results suggest that the model is very sensitive to the amount of auxin

in the meristem. Too low auxin synthesis (change by more than 10%) or too high degradation (change by more than 20%) stops the formation of patterns. This can be noted by observing $\alpha_a, \beta_a$ curves.

2. The increase of auxin transport (passive $\gamma_d$ or active $\gamma_a$) decreases $f$. More efficient transport depletes the auxin faster from the meristem surface, increasing the inhibitory range of the primordium and increasing the time between the initiation of the consecutive primordia.

3. The increase of PIN background insertion into the membrane $\alpha_p$ (or decrease of PIN removal $\beta_p$) increases the strength of the active transport, therefore it decreases $f$. This can be observed in $\alpha_p, \beta_p$ curves.

4. The increase of competence zone size $CZ_{size}$ leads to an increase of $f$. This observation is consistent with biology and other inhibitory field model predictions [5, 6].

5. The increase of the primordium initiation threshold $\omega$ decreases $f$.

The sensitivity analysis of 1D flux-based dynamic system shows that $i$) the patterning capabilities of the model are kept for a range of parameter values $ii$) auxin synthesis, auxin decay and the primordia initiation threshold have the the biggest impact on the frequency $f$.

## 1.3 Sensitivity of the 2D flux-based polarization model to noise

The proposed mechanism of dynamic patterning depends on the detection of the locations where the auxin concentration exceeds a given threshold $\omega$. The cells at this location differentiate and become sinks. We tested how sensitive this model is to the random auxin fluctuations in the system. This was achieved by i) adding noise to the already established auxin patterns and checking at what level the noise was able to perturb the PIN polarization pattern (in a non-growing tissue), ii) change the primordia initiation threshold $\omega$ in each cell by adding a noise with controlled amplitude to check the stability of primordium formation during development. The tests were performed on a system that was able to generate spiral phyllotaxy (equations and parameters were set according to Simulation 9A).

Let $m_{i \to j}$ be the membrane in cell $i$ at the interface with cell $j$ and $M$ denote the set of all the membrane interfaces in the considered tissue. For each time $t$, we denote $P_m(t)$ the concentration of PIN in this $m \in M$ at time $t$.

In the first case, the arbitrary frame of Simulation 9A was chosen. This frame was describing system state at reference time $t_0$. Then auxin concentration was modified in each cell by adding a white noise of amplitude $\delta = k5\%$ (for $k \in \{1, 2, .., 5\}$). Then the system of equations describing the PIN and auxin dynamics was resolved, until a new stable state was reached. Then, the new PIN distributions $P'_m(t_0)$ were compared with the initial distributions $P_m(t_0)$ for every $m \in M$. The comparision between the simulation was done by comparing the average difference of PIN concentration $s = (1/|M|) \sum_{m \in M} |P'_m(t_0) - P_m(t_0)| / P_m(t_0)$,

where $|M|$ denotes the size of $M$. As a result, we could observe that, the average difference of PIN concentration $s$ was increasing with increasing values of $k$. However, for each $k$, $s$ was found less than 5% and the system reached a state that was close to the original configuration. This showed that the flux-based model is fairly robust to auxin level perturbation throughout the meristem.

In the second case the simulation was performed exactly as in Simulation 9A, with one exception: the primordium initiation threshold $\omega_i$ was defined in every cell $i$ as a mean threshold $\omega$ modified by an additive white noise of amplitude $\delta = \omega 2k\%$ ($k \in \{1, 2, .., 5\}$). This threshold noise was modified at every step of the simulation. By increasing the noise with a fixed primordium initiation threshold, we structurally change the speed at which primordia are detected, which results in a change in the phyllotactic pattern. To avoid this situation, the primordium initiation threshold $\omega$ was increased by $\omega\delta$. The results showed that the system is robust for low noise amplitudes: for $k \in \{1, 2\}$ the system was able to reproduce the spiral pattern with minor errors. These errors corresponded to occasional changes in spiral orientation (chirality) during development. The system was fragile for higher noise ($k > 2$). In such cases, spiral patterns were not maintained. This suggests that flux-based polarization may require an additional mechanisms to achieve high robust behaviour.

# 2 Simulations

## 2.1 The basic system of equations

The basic system of equations equations, which we use in the simulations are defined as in the article:

$$\frac{\partial a_i}{\partial t} = -\sum_{n \in N_i} \frac{S_{i \to n}}{V_i} J_{i \to n} + \alpha_a - \beta_a a_i \tag{1}$$

$$\frac{\partial p_{i,n}}{\partial t} = \Phi\left(J_{i \to n}\right) + \alpha_p - \beta_p p_{i,n} \tag{2}$$

$$J_{i \to n} = \gamma_a\left(a_i p_{i,n} - a_n p_{n,i}\right) + \gamma_d\left(a_i - a_n\right) \tag{3}$$

$$\Phi_L(x) = \begin{cases} \kappa x & x \geq 0 \\ 0 & x < 0 \end{cases} \tag{4}$$

$$\Phi_C(x) = \begin{cases} \kappa x^2 & x \geq 0 \\ 0 & x < 0 \end{cases} \tag{5}$$

In the forthcoming sections we report which basic equation is used and which is modified for a particular simulation. The parameters for the system can be found in the supplementary Table 1.

## 2.2 Figure 2A

**Specification:** The cells which belong to $Si$ were selected as described in the main article. The simulation was run for a fixed amount of steps. The system reached stability before the last step.

**Model:** We use equations 2, 4, we redefine 1, 3:

$$\frac{\partial a_i}{\partial t} = \left( - \sum_{n \in N_i} J_{i \to n} + \alpha_a - \beta_a a_i \right) [i \notin Si]$$

$$J_{i \to n} = \gamma_a \left( a_i p_{i,n} - a_n p_{n,i} \right),$$

And we assume that $\Phi = \Phi_L$.

## 2.3 Figure 2B

**Model:** The simulation differs from 2.2 only by modifying equation 1 in the following way:

$$\frac{\partial a_i}{\partial t} = - \sum_{n \in N_i} J_{i \to n} + \alpha_a - \left( \beta_a + \beta'_a [i \notin Si] \right) a_i$$

All the parameters are exactly the same and can be found in the Table 1.

## 2.4 Figure 3A

**Specification:** The cells which belong to $Si$ were selected and the simulation was run for a fixed amount of steps. The system reached stability before the last step.

**Model:** We use equations 2, 3, 4, we redefine 1:

$$\frac{\partial a_i}{\partial t} = \left( - \sum_{n \in N_i} J_{i \to n} + \alpha_a - \beta_a a_i \right) [i \notin Si]$$

And we assume that $\Phi = \Phi_L$.

## 2.5 Figure 3B

**Specification:** The cells which belong to $Si$ were selected and the simulation was run for fixed amount of steps. The system was stable after the last step. The figure presented in the text is not the last step of the simulation. The system still develops and creates more complex vein pattern (with loops). This evolution can be observed on the supporting movie.

**Model:** We use equations 2, 3, 5, we redefine 1:

$$\frac{\partial a_i}{\partial t} = \left( - \sum_{n \in N_i} J_{i \to n} + \alpha_a - \beta_a a_i \right) [i \notin Si]$$

And we assume that $\Phi = \Phi_C$.

## 2.6 Figure 4A-4D

**Model:** We use equations 2, 3, 4, we redefine 1:

$$\frac{\partial a_i}{\partial t} = \left( - \sum_{n \in N_i} J_{i \to n} + \alpha_a - \beta_a a_i \right) [i \notin Si]$$

And we assume that $\Phi = \Phi_L$.

## 2.7 Figure 5A-5F

**Model:** We use equations 2, 3, 4, we redefine 1:

$$\frac{\partial a_i}{\partial t} = \left( - \sum_{n \in N_i} J_{i \to n} + \alpha_a - \beta_a a_i \right) [i \notin Pr]$$

And we assume that $\Phi = \Phi_L$.

## 2.8 Figure 7A-7B

**Specification:** The simulation was run in two variants: with and without a centre. In case of " the simulation with a centre a subset of cells which belong to $Cz$ was chosen. These cells were degrading the auxin. The initial geometry of the cells was acquired from confocal images. The simulation was run for 60 steps until it reached a stable state with a visible auxin maximum in the place of the future initium.

**Model:** We use equations 2, 3, 4, we redefine 1:

$$\frac{\partial a_i}{\partial t} = \left( - \sum_{n \in N_i} \frac{S_{i \to n}}{V_i} J_{i \to n} + \alpha_a - (\beta_a + \beta'_a [i \notin Cz]) a_i \right) [i \notin Pr]$$

And we assume that $\Phi = \Phi_L$.

## 2.9 Figure 9A-9E

**Specification:** To simulate the influence of old primordia, each primordium 'leaving the virtual meristem through growth tagged the closest neighbour cell with a special cell identity (yellow dots).. This property was propagated over a given time. The cells tagged as such were acting as sinks but the sink strength was gradually decreasing with time

**Model:** We use equations 2, 3, 4, we redefine 1:

$$\frac{\partial a_i}{\partial t} = \left( - \sum_{n \in N_i} \frac{S_{i \to n}}{V_i} J_{i \to n} + \alpha_a - (\beta_a + \beta'_a [i \notin Cz]) a_i \right) [i \notin Pr]$$

And we assume that $\Phi = \Phi_L$.

## 2.10 Figure 9G-9I

**Specification:** Cells with white dots are simulating old primordia and they belong to $Si$ set, cells with black dots are L1 cells and they belong to $L1$ set, the cell with a blue dot is a new primordium and belongs to the $Pr$ set (note: it belongs to $L1$ set as well). We assume that the L1 cells are separated from the inner cells except for the primordium cell. This cell is allowed to exchange the auxin with both L1 and inner cells. The feedback from flux on PIN polarisation in L1 and inner cells is different and it is modelled with a change in$\Phi$ function. Also, the cells in L1 layer produce much more auxin than inner layer cells.

**Model:** We use equations 3, 4, 5 we redefine 1, 2:

For cells $i$ such as $i \in L1$:

$$\frac{\partial a_i}{\partial t} = \left( - \sum_{n \in N_i} \frac{S_{i \to n}}{V_i} J_{i \to n} \left[ n \in L1 \vee i \in Pr \right] + (\alpha_a + \alpha_{L1}) - \beta_a a_i \right) \left[ i \notin Si \right]$$

$$\frac{\partial p_{i,j}}{\partial t} = \Phi_L \left( J_{i \to j} \right) \left[ j \in L1 \right] + \Phi_L \left( J_{i \to j} \right) \left[ j \notin L1 \right] + \alpha_p - \beta_p p_{i,j}$$

For cells $i$ such as $i \notin L1$:

$$\frac{\partial a_i}{\partial t} = \left( - \sum_{n \in N_i} \frac{S_{i \to n}}{V_i} J_{i \to n} \left[ n \notin L1 \vee i \in Pr \right] + \alpha_a - \beta_a a_i \right) \left[ i \notin Si \right]$$

$$\frac{\partial p_{i,j}}{\partial t} = \Phi_C \left( J_{i \to j} \right) + \alpha_p - \beta_p p_{i,j}$$

**Parameters:** $\alpha_{_{L1}} = 0.3$

## 2.11 Figure 10C

**Specification:** The simulation was run until the system reached a stable state. In this state an auxin maximum was established in the root apex.

We use equations 3, 2, 5 we redefine 1:

$$\frac{\partial a_i}{\partial t} = \left( - \sum_{n \in N_i} \frac{S_{i \to n}}{V_i} J_{i \to n} + \alpha_a - \beta_a a_i \right) \left[ i \notin Si \right]$$

And we assume that $\Phi = \Phi_C$.

**Initial conditions:** $\forall i \notin Si.a_i = 0.3; \forall i \in Si.a_i = 0.0; p_{i.j} = 1.0 \, if \, PIN \, exists \, in \, vivo \, else \, 0$

| Parameter | 2A | 2B | 3A | 3B | 4A-D | 5A-F | 7A-B | 9A-E | 9G-I | 10C |
|---|---|---|---|---|---|---|---|---|---|---|
| $\alpha_a$ | 0.1 | 0.1 | 0.1 | 0.1 | 0.1 | 0.1 | 0.1 | 0.3 | 0.01* | 0.0 |
| $\beta_a$ | 0.01 | 0.01 | 0.01 | 0.01 | 0.01 | 0.01 | 0.01 | 0.03 | 0.01 | 0.0 |
| $\beta'_a$ | - | 0.1 | - | - | - | - | -/0.08 | 0.06 | - | - |
| $\alpha_p$ | 0.1 | 0.1 | 0.1 | 0.1 | 0.1 | 0.1 | 0.1 | 0.1 | 0.1 | 0.0 |
| $\beta_p$ | 0.05 | 0.05 | 0.01 | 0.01 | 0.01 | 0.05 | 0.01 | 0.01 | 0.01 | 0.5 |
| $\gamma_a$ | 0.1 | 0.1 | 0.1 | 0.1 | 0.1 | 0.1 | 0.1 | 0.125 | 0.1 | 100.0 |
| $\gamma_d$ | - | - | 0.03 | 0.03 | 0.03 | 0.001 | 0.03 | 0.03 | 0.03 | 0.001 |
| $\Phi$ | L | L | L | C | L | L | L | L | L+C | C |
| $\kappa_L$ | 0.15 | 0.15 | 0.2 | - | 1.3/1.5/1.7/2.0 | 0.15 | 0.18 | 0.11 | 0.09 | - |
| $\kappa_C$ | - | - | - | 1.3 | - | - | - | - | 1.1 | 0.2 |
| $h$ | 2.2 | 2.2 | 1.0 | 0.025 | 1.0 | 1.0 | 0.25 | 1.0 | 1.0 | 0.1 |
| $\epsilon_0$ | - | - | - | - | - | 0.01 | - | 0.01 | - | - |
| init $a_i$ | 0.0 | 0.0 | 0.0 | 0.0 | 0.0 | 0.0 | 0.0 | 0.0 | 0.0 | 0.3 |
| init $p_{i,j}$ | $\frac{\alpha_p}{\beta_p}$ | $\frac{\alpha_p}{\beta_p}$ | $\frac{\alpha_p}{\beta_p}$ | $\frac{\alpha_p}{\beta_p}$ | $\frac{\alpha_p}{\beta_p}$ | $\frac{\alpha_p}{\beta_p}$ | $\frac{\alpha_p}{\beta_p}$ | $\frac{\alpha_p}{\beta_p}$ | $\frac{\alpha_p}{\beta_p}$ | * |
| PIN max | - | - | 2.0 | 1.0 | 2.0 | - | 2.0 | 2.0 | 2.0 | 1.0 |
| snapshots | 100 | 100 | 60 | 240 | 120 | 400 | 60 + 60* | * | 30 + 80* | 2500 |
| steps | 100 | 100 | 10 | 400 | 10 | - | 10 | - | 10 | 10 |
| $[a_{min}, a_{max}]$ | - | - | [0, 5] | [0, 5] | [0, 5] | - | [0, 4.8] | [0, 6.8] | [0, 12] | [0, 1] |
| $p_{mid}$ | - | - | 0.94 | 0.94 | 0.94 | - | 0.9 | 0.7 | 0.6 | 0.5 |

Tab. 1: The simulation parameters. The "*" is used when the parameter is changed in complex way (which is explained in details in the text), the "/" means alternative values used in simulation and "-" means that the value is not included in the experiment equations. The units for the parameters were specified in the main article, however to adjust them to cellular world all the $m$ should be exchanged by $\mu m$ and $mol$ by $\mu mol$.

## References

[1] Iverson KE (1962) A Programming Language.1. John Wiley & Sons.

[2] Knuth DE (1992) Two notes on notation. Am Math Monthly 99:403–422. doi:10.2307/2325085.

[3] Jones E, Oliphant T, Peterson P (2001). Scipy: Open source scientific tools for python.

[4] Hindmarsh AC (1983) ODEPACK, A Systematized Collection of ODE Solvers , R. S. Stepleman et al. (eds.), North-Holland, Amsterdam, (vol. 1 of ), pp. 55-64., volume 1 of *IMACS Transactions on Scientific Computation*. North-Holland Amsterdam, 55–64 pp.

[5] Douady S, Couder Y (1996) Phyllotaxis as a dynamical self organizing process part ii: The spontaneous formation of a periodicity and the coexistence of spiral and whorled patterns. Journal of Theoretical Biology 178:275–294. doi:10.1006/jtbi.1996.0025.

[6] Smith R, Kuhlemeier C, Prusinkiewicz P (2006) Inhibition fields for phyllotactic pattern formation: a simulation study. Canadian Journal of Botany 84(11):1635–1649.
